# Amplifying cancer immunity: AMPK activators and gammadelta T cells unveiled

**DOI:** 10.17179/excli2024-7784

**Published:** 2024-10-29

**Authors:** Firoz Anwar, Fahad A. Al-Abbasi, Naif Abdullaha R. Almalki, Sultan Alhayyani, Amita Verma, Vikas Kumar

**Affiliations:** 1 Department of Biochemistry, Faculty of Science, King Abdulaziz University, Jeddah, Saudi Arabia; 2 Experimental Biochemistry Unit, King Fahad Medical Research Center, King Abdulaziz University, Jeddah, Saudi Arabia; 3 Department of Chemistry, College of Sciences and Arts, King Abdulaziz University, Rabigh, Saudi Arabia; 4 Bioorganic and Medicinal Chemistry Research Laboratory, Department of Pharmaceutical Sciences, Sam Higginbottom University of Agriculture, Technology and Sciences, Prayagraj, Uttar Pradesh, India; 5 Natural Product Drug Discovery Laboratory, Department of Pharmaceutical Sciences, Sam Higginbottom University of Agriculture, Technology and Sciences, Prayagraj, Uttar Pradesh, India; 6 University Center for Research and Development, Chandigarh University, Gharuan 140413, Punjab, India

## ⁯⁯⁯

γδ-T cells, which are part of an active immunity, have impressive anticancer capabilities and are able to broadly target tumors without requiring a patient-specific antigen or a specific type of leukocyte antigen on the cancer patient (Dai et al., 2024[3]; Mensurado et al., 2023[6]), making γδ T cells to sense the altered stress signals in prevalent in transformed cells (Sebestyen et al., 2020[8]). Though we are still in juvenile phase to establish the precise mechanism governing and recognizing the stressed cells. Among human γδ T cells, Vγ9Vδ2 T cells stand out as the most abundant subset. These cells identify a protein complex of butyrophilin 2A1 (BTN2A1) and BTN3A1 which are widely expressed on cell surface activated by phosphoantigens profusely produced by tumor cells (Mamedov et al., 2023[5]), making them promising agents for the advancement of tumor immunotherapy. Innate immune response mediated by γδ T cell receptor, in cancer patients demonstrates broad reactivity but lacks the antigenic specificity seen in αβ TCR. Among γδ T cells, Vγ9Vδ2 T cells, which are specific to the BTN2A1-BTN3A1-BTN3A2 complex, have shown significant potential in antitumor immunity. Elevated expression of BTN3A1 in tumor cells regulated by the mevalonate pathway facilitates the recruitment of BTN2A1 thereby activating the Vγ9Vδ2 T cells. This interaction enhances understanding of the complex signaling mechanism involved in γδ T cell-mediated tumor recognition and cytotoxicity. Many insight dynamics between cancer cells and Vγ9Vδ2 T cells are yet to be deciphered due to poor understanding, making it more challenging for glioma scientists (Close et al., 2020[2]; Thomas et al., 2024[9]).

Mamedov et al. employed CRISPR technology to unveil the genes governing the cancer-killing capabilities of human Vγ9Vδ2 T cells, on Daudi-Cas9 cell line expressing Cas9. Cells were treated with Zoledronic acid and were cultured with primary Vγ9Vδ2 T cells from healthy donors to examine the live Daudi cells. Important regulators were identified in the interaction between cancer cells and Vγ9Vδ2 T cells responsible for enhanced survival rates via genes related to the butyrophilin complex, mevalonate pathway enzymes, transporter genes, gene activators, and surface proteins (Rigau et al., 2020[7]), providing significant insight for new strategies in cancer therapeutics. This breakthrough holds significant promise for advancing cancer treatment strategies. Metabolic pathways fundamental for survival during co-culture, with purine metabolism, oxidative phosphorylation and TCA cycle exhibited notable enrichment via gene set enrichment analysis (GSEA). A genome-wide CRISPR screen was conducted to identify key regulatory genes involved in modulating the expression and surface localization of BTN3A, aiming to elucidate the molecular pathways that govern its cell surface levels. The cytotoxic function of Vγ9Vδ2 T cells on tumor cells is fundamentally associated with BTN3A levels on the cell surface. Significant expression FDR < 0.01 of BTN3A was observed in co-culture cells, deciphering the fundamental role of BTN3A in modulating the effectiveness of Vγ9Vδ2 T cell-mediated killing (Yuan et al., 2024[11]). 

The Vγ9Vδ2 TCR-BTN2A1/3A1 interaction is a function of phosphoantigen concentration, and is highly influenced by plays through mevalonate pathway (Yuan et al., 2023[10]), however its surface expression in excess was yet to be deciphered. The study reveals that the knockout of the mevalonate pathway enzyme FDPS (farnesyl diphosphate synthase) along with enzymes involved in pathways such as *de novo* purine synthesis and Fe-S cluster synthesis results in increased BTN3A levels on the cell surface and improves the antitumor activity and efficiency. Furthermore, the adenosine monophosphate (AMP), regulates AMPK and ATP synthesis from *de novo* purine synthesis pathway, can further influence BTN3A and its impact on tumor cell destruction.

The result assay further indicates a reciprocal regulation by IRF1 and ZNF217, with interplay between oxidative phosphorylation and BTN3A expression. It was observed that no association between disturbance in the electron transport chain complex I contraindicate earlier studies that changes in ATP synthesis and the NADH:NAD+ ratio influence BTN3A expression. Experiments have demonstrated that BTN3A expression relies on energy; heightened glucose levels and the inhibition of glycolysis or specific complexes raise surface BTN3A levels. They tested whether metformin, a secondary activator of AMPK, slightly increased the expression of BTN3A on the cell surface in WT Daudi-Cas9 cells, which was confirmed by direct activators, C991 and A-769662. Interestingly, breast and colon cancer organoids derived from patients treated with AMPK activators also demonstrated improved levels of BTN3A on the cell surface. It's worth noting that eliminating AMPK without exposing the cells to stress conditions did not lead to any alterations in BTN3A.

Natural compounds as AMPK activators include Metformin, Resveratrol, Salicylate. Small drug molecules like Thienopyridone, 991 (ex229), PF-06409577 (Compound 7), Compound-2/Compound-13, PT-1, MT 63-78 (Debio 0930), MT47-100, JJO1 (Guigas and Viollet, 2016[4]) are already in use for various metabolic disorders. Their new mechanistic approach (Anagnostakis and Piperi, 2023[1]) via empowering γδ T cells can open new avenues in retarding the cases of glioma either by inhibiting glioma growth, inducing apoptosis, preventing angiogenesis or even changing the microenvironment that can inhibit the stress on neurons exploring the potential synergies of combining natural compounds with conventional glioma treatments with minimum adverse effects.

Drugs activating AMPK can lead to high expression of BTN immunoglobulin on cancer cells including gliomas with high chances of Vγ9Vδ2 T cell receptor-mediated cytotoxicity suggesting new possibilities to boast γδ T cell anticancer efficacy. Transcription, post translation modifications and membrane alterations were already known to initiate γδ T cell responses, but small drug molecules that can be able to cross the blood brain barrier give new hope particularly related to ATP production via metabolic alteration by the drug molecules without putting stress on tumor cells. 

The limitations surrounding above hypothesis are primarily due to the incomplete understanding of the precise mechanism by which γδ T cells recognize and attack stress tumor cells. While γδ T cells, particularly the Vγ9Vδ2 subset, are known for their broad anticancer activity, current knowledge of how they sense altered stress signals and the complex interaction between the mevalonate pathway and other metabolic regulators remains underdeveloped. Despite the potential by AMPK activators as a new therapy targeting γδ T cell, further research is required to translate these findings into practical drug discovery strategies. 

## Notes

Firoz Anwar and Vikas Kumar (Natural Product Drug Discovery Laboratory, Department of Pharmaceutical Sciences, Sam Higginbottom University of Agriculture, Technology and Sciences, Prayagraj, Uttar Pradesh, India; University Center for Research and Development, Chandigarh University, Gharuan 140413, Punjab, India; E-mail: phvikas@gmail.com) contributed equally as corresponding author.

## Declaration

### Consent of publication 

None.

### Acknowledgments

The authors extend their appreciation to the King Abdulaziz University, Jeddah, Saudi Arabia.

### Conflict of interest

The authors declare no conflict of interest, financial or otherwise.
